# “Smartphone as an educational tool” the perception of dental faculty members of all the dental colleges of Khyber Pakhtunkhwa - Pakistan

**DOI:** 10.1186/s12909-023-04093-8

**Published:** 2023-02-20

**Authors:** Syed Muhammad Junaid, Brekhna Jamil, Muhammad Adnan Khan, Zainab Akbar, Sana Shah, Natasha Nadeem, Anum Nadeem

**Affiliations:** 1 Rehman College of Dentistry, Peshawar, Pakistan; 2 Khyber Medical University, Peshawar, Pakistan; 3 Northwest School of Medicine, Peshawar, Pakistan

**Keywords:** Attitude, Faculty Memebers, Medical Education, Perceptions, Smartphones, Teaching tool

## Abstract

**Background:**

With the increasing advancement in the field of information technology, it’s about time we realize that our future will be shaped by this field. With more and more people using smartphones, we need to adapt them to the medical field. Already many advancements in medical field are done thanks to the advancement of computer science. But we need to implement this into our teaching and learning as well. Almost all students and faculty members use smartphones in one way or another if we can utilize the smartphone to enhance the learning opportunities for our medical students, it would greatly benefit them. But before the implementation, we need to find out if our faculty is willing to adopt this technology. The objective of this study is to find out what are the perceptions of dental faculty members about using a smartphone as a teaching tool.

**Methodology:**

A validated questionnaire was distributed among the faculty members of all the dental colleges of KPK. The questionnaire had 2 sections. First one contains information regarding the demographics. The second one had questions related to the faculty members’ perception regarding using a smartphone as a teaching tool.

**Results:**

The results of our study showed that the faculty (Mean 2.08) had positive perceptions regarding using a smartphone as a teaching tool.

**Conclusion:**

Most of the Dental Faculty members of KPK agree that smartphone can be used as a teaching tool, and it can have better outcomes if proper applications and teaching strategies are used.

**Supplementary Information:**

The online version contains supplementary material available at 10.1186/s12909-023-04093-8.

## Introduction

### Background

The dawn of the new century came with the fruits of advancement in technology. Smartphones have multitude of features and have started to come at the forefront of keeping up with the rapid advancements in the field of science and technology. Due to their ease of use and extensive functionality, smartphones are getting attention in the healthcare sector. People are beginning to use them as devices not just to stay connected but consume knowledge and spend their free time with them. In order to keep connected with the developing world, a smartphone is a necessity and medical educationists around the world have realized this importance [1].

Digital technologies have been used not only in education but also in clinical scenarios with clinical success [2]. Yet there are certain individuals who don’t use this technology at all. Their use is limited to call and short message service specially in older adults. The limitations could be because of many reasons, from lacking the necessary knowledge or finding it difficult to learn the functions [3].

Smartphones help students and teachers to access and process knowledge on the go [4]. The boundaries of the classroom are no longer a prerequisite for learning. The place as well as the roles of the learners along with the teaching methods are changed [5].

Traditionally, Teacher centered learning has been practiced but recently, the tilt is shifting toward a student-centered learning style [6]. In student centered learning the student is in the driving seat, they decide about how, what and when they learn. Their input is highly valued and drives the learning process [7]. The use of technology in Dentistry is not new. It was first shown in 1971 [5]. A scoping review evaluated the use of WhatsApp in dental education. They reviewed studies between 2016 and 2020 and concluded that WhatsApp is being used by dental students to improve communication and enhance their learning by actively engaging in discussions via the platform [8].

Studies have shown that various universities around the world are now implementing the use of smartphones into their teaching and learning strategies. They revealed that implementing such techniques improved the student cooperation and integration [9]. From a strictly medical point of view different studies have shown us the use of smartphones in academic teaching and learning. The Dental students in Australia accessed their course via their smartphones. Furthermore, they also searched for learning material online, took important notes and snaps of important slides.^(1)^ The medical teachers and learners in Canada involved the learners in managing and taking care of their patients via their smartphones [10]. Implementing newer technologies into medical and dental curricula is also possible. By implementing such tools not only learning can be enhanced but other skills can also be polished like teamwork, collaboration and problem-solving [11].

Despite all these advantages and benefits of technology, there are still certain drawbacks to the use of smartphones. Students can take advantage of this situation and use their smartphones during class time to surf the internet or text their friends and waste their precious time. They are a very easy distractor for students [12][13].

### Purpose of the study

Very few studies have been carried out to assess the teacher’s perception about using smartphones as a learning tool. The majority of the literature talks about students or learners’ perspectives without taking into consideration of instructor’s perception. The present study accessed the teacher’s perception about the use of smartphones as an educational tool among the dental teaching faculty of Khyber Pakhtunkwa.

### Objective of the study

The objective of this study is:

- To determine dental faculty members’ perceptions of using smartphones as an educational tool.

## Methodology

### Materials and methods

#### Study setting

The study was conducted in all the dental colleges of KPK. Both private and public sector colleges were included in the study. The Study included the following colleges.

1. Abbottabad International Medical & Dental College, Abbottabad.
2. Bacha Khan College of Dentistry, Mardan.
3. Dental Section, Ayub Medical College, Abbottabad.
4. Frontier Medical & Dental College, Abbottabad.
5. KMU Institute of Dental Sciences, Kohat.
6. Khyber College of Dentistry, Peshawar.
7. Peshawar Dental College, Peshawar.
8. Rehman College of Dentistry, Peshawar.
9. Saidu College of Dentistry, Saidu Sharif.
10. Sardar Begum Dental College, Peshawar.
11. Women Medical & Dental College, Abbottabad.

#### Study design and type

It was a quantitative cross-sectional descriptive survey carried out to collect the responses of the participants on a specifically designed data collection instrument.

### Approval of the study

The approval for this study was obtained in 116th meeting of the KMU ASRB held on 31st March 2022. (Annexure I).

### Sample selection

#### Inclusion criteria

All the dental teaching faculty of KPK. All basic and clinical subjects including.

#### Exclusion criteria

Any dental faculty member not using a smartphone and who does not wish to be part of the study.

### Sample size calculation

Target Population was selected by calculating the faculty required in all dental colleges of KP. OpenEPI was used to calculate the sample size with a 95% Confidence Level. It came out to be 267. with 20% attrition, it was calculated to be 320.

### Data collection instrument

A preformed and validated questionnaire was used to collect the data [14]. The study instrument has been beforehand tested and was considered reliable with Cronbach’s Alpha score α = 0.95.

### Data collection procedure

After taking permission from all the dental colleges of KPK for data collection, questionnaire was shared among the dental faculty members working at different dental colleges of KPK. There were two parts of this survey. Part one included the independent variables; the questions were related to demography. Dependent variable was represented by the second part of the questionnaire, which consisted of fourteen [14] items relating to the perception of the teacher towards using of smartphones as an educational device. We utilized Likert Scale with five points for the measurement.

### Data analysis procedure

Data was normally distributed, mean and percentages were used to analyze it. The associations among demographic elements and the achieved scores were calculated using Chi-square tests; where two variables were used student T-test was applied. SPSS version 26 was used to analyze the data. Unanswered questions were indicated by using absolute values with percentages. The level of significance was kept at < 0.05).

### Ethical concerns

Ethical approval was obtained as a formal permission from all the dental colleges of KPK. A paper-based questionnaire form was used in this research. Moreover, a verbal explanation was given to each potential participant describing the questionnaire in simple language and assuring confidentiality and volunteer participation. Also, verbal assurance was given, that only the result finding will be utilized and published for the said purpose rather than marketing and the identity of the participants will be kept confidential. Also, the names of the colleges were not shown in the results.

## Results

### Demographic parameters

**Fig. 1:**
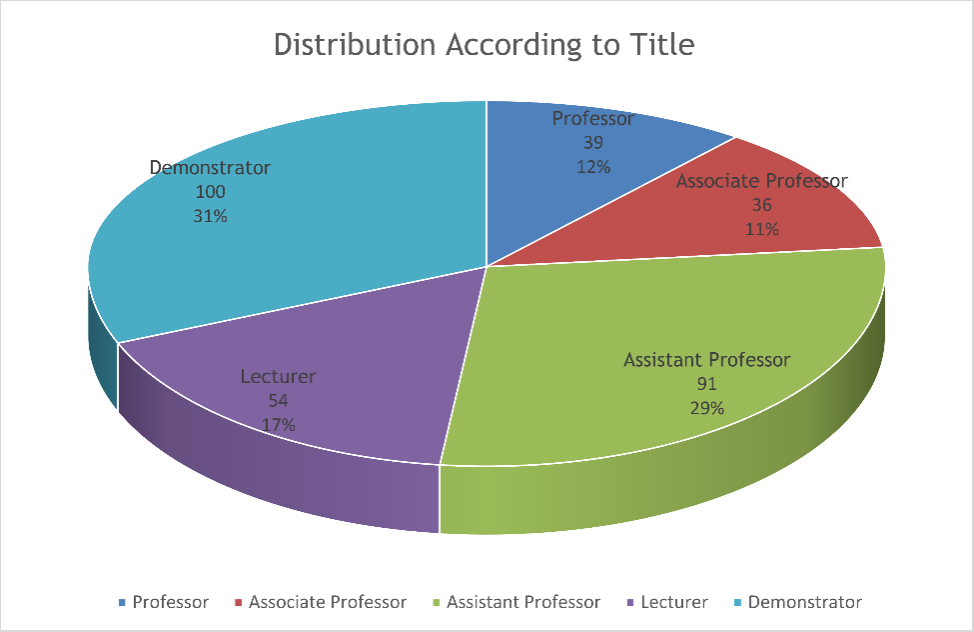
Distribution of the Study Participant saccording to Title

A total of 320 dental faculty members answered the questionnaire. The number varied among the dental colleges, as the faculty of each college is different depending upon the number of students in that college. According to title the questionnaire was answered by 39 Professors, 36 Associate Professors, 91 Assistant Professors, 54 Lecturers and 100 Demonstrators. Figure 1 shows us the distribution in a graphical manner.

**Fig. 2:**
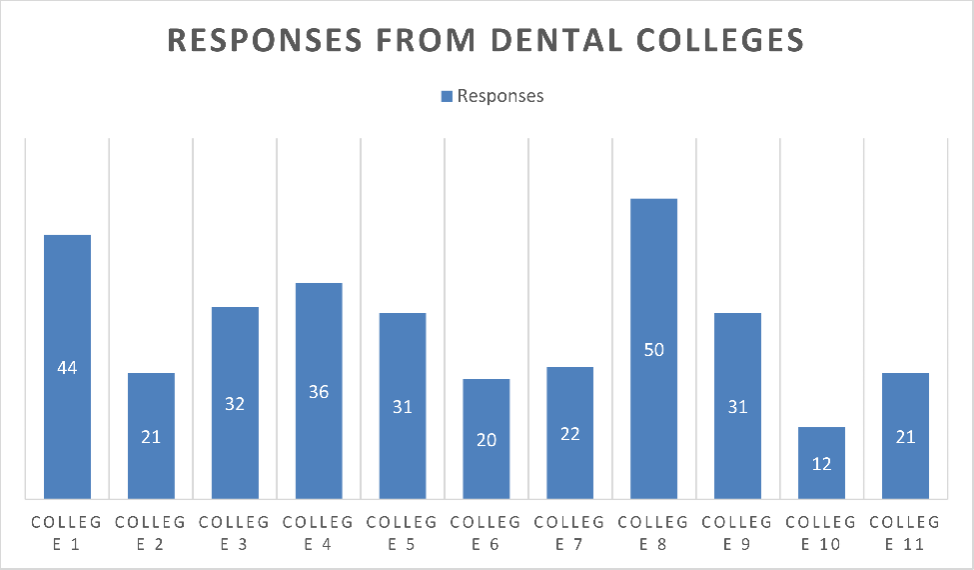
Responses from dental colleges

Figure 2 shows us that eleven Dental Colleges are currently offering the Bachelor’s of Dental Surgery (BDS) degree program in Khyber Pakhtukhwa. They all were included in this study. The responses we collected were not equal. It was because of the reason that faculty members in different colleges are added according to the number of students each college is permitted to admit and teach. So, the responses were different, the following chart explains the results obtained from different colleges.

**Fig. 3:**
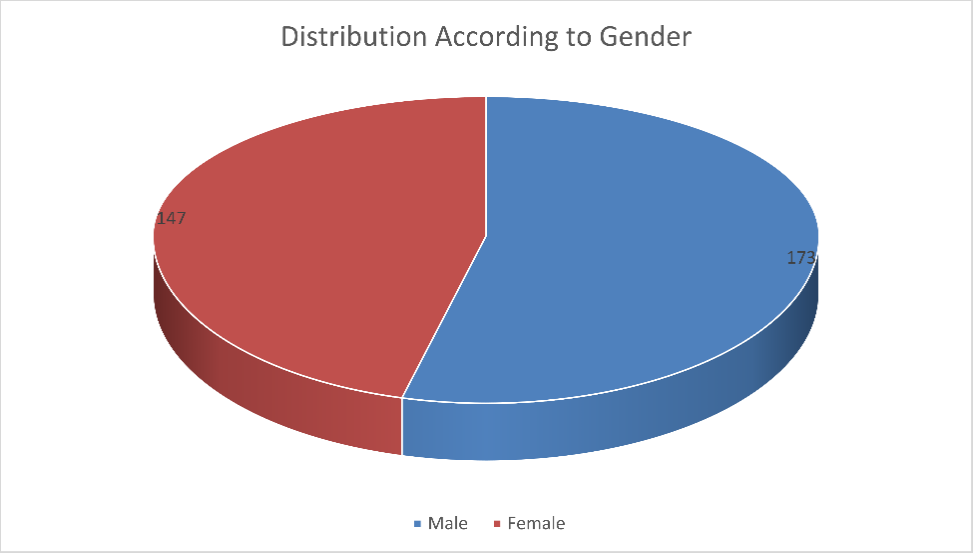
Distribution according to Gender

According to Gender, we have a distribution of 173 Males and 147 Females who have responded to the questionnaire and submitted it back. This has been shown to us graphically in Fig. 3. This represents all the dental colleges of KPK. This shows us that 46% of the respondents were Females and 54% of the respondents were Males.

**Fig. 4:**
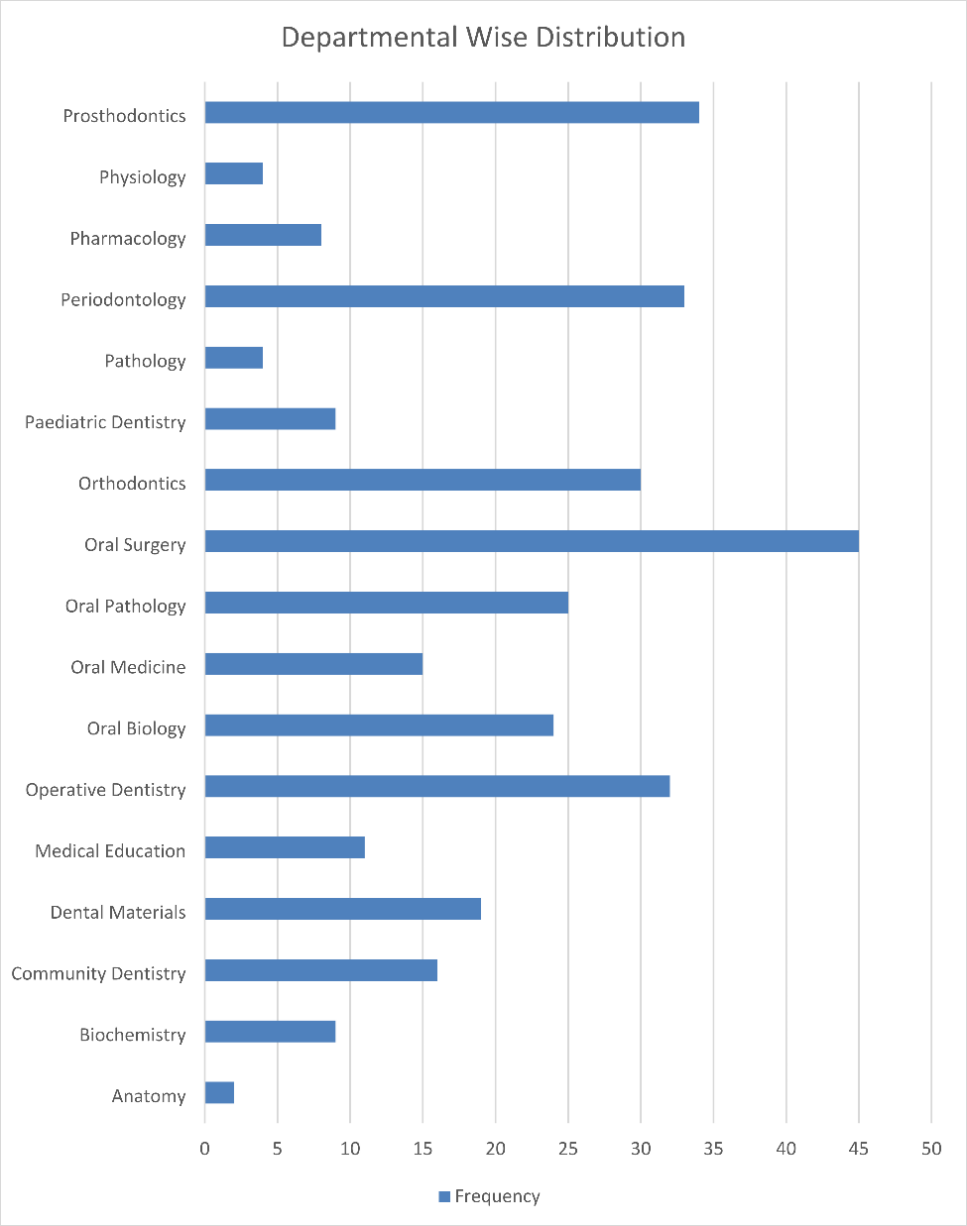
Departmental Distribution of the Data

According to Departments we have a bar graph in Fig. 4 depicting different departments who answered our questionnaire.

The above data shows us that Anatomy Department had the lowest return of the questionnaire, comprising of only 2 and Oral Surgery Department had the most filled questionnaires returned which stands at 45.

According to the experience, we divided it into 3 categories in the questionnaire namely, less than 5 years, 5 to 10 years, and more than 10 years of experience. The results of which are as follows.

**Fig. 5:**
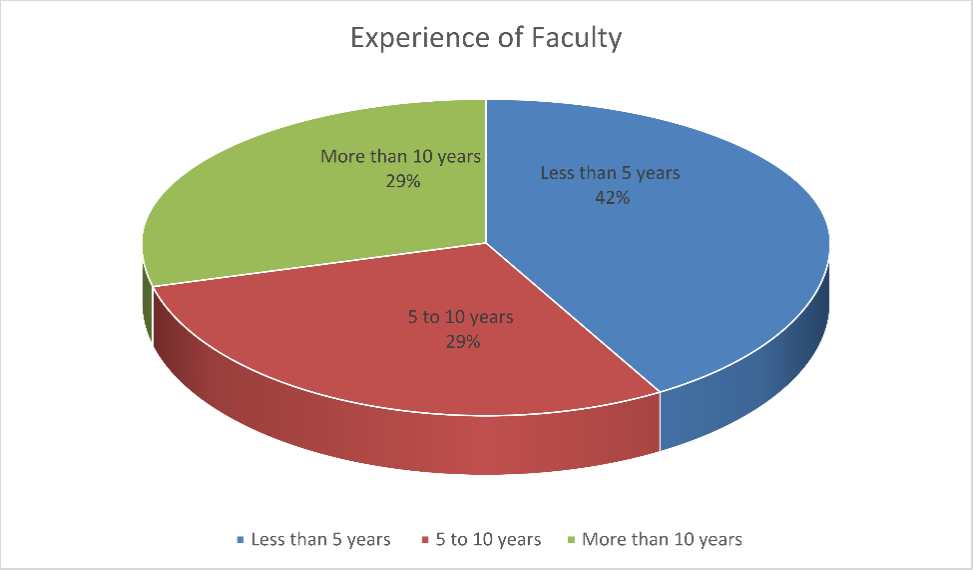
Experience of Faculty

This pie chart in Fig. 5 shows that 42% of the respondents were having an experience of less than 5 years. However, 5 to 10 years and more than 10 years comprised of about 29% each.

**Fig. 6:**
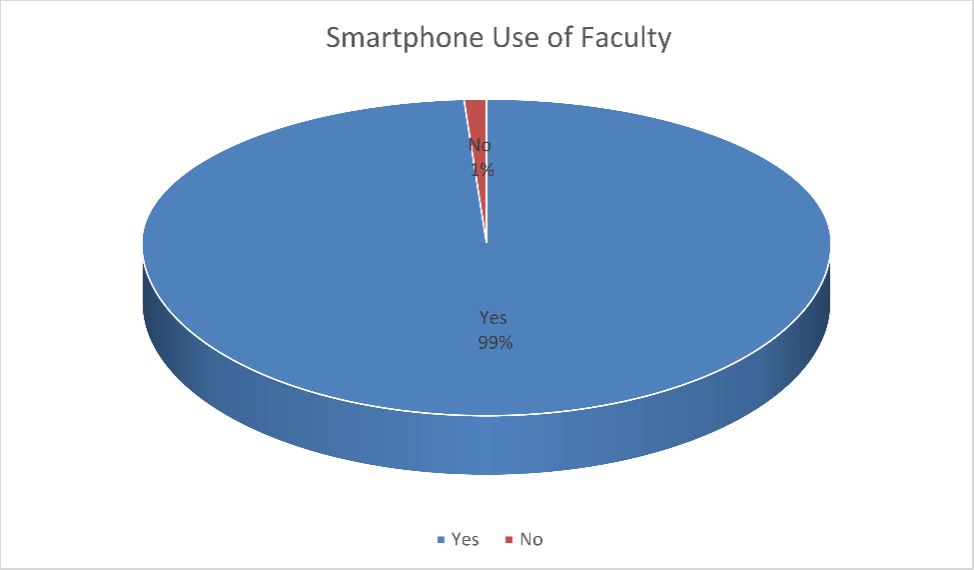
Smartphone use of Faculty

Among the faculty when questioned about whether they used a smartphone or not. 316 of the respondents said they used a smartphone and only 4 of the respondents said that they didn’t use a smartphone. Hence, they didn’t qualify to fill the rest of the questionnaire. Pie chart in Fig. 6 is showing us that.

### The perceptions of teaching faculty members towards using the smartphone as a teaching tool

**Table 1:** The perception of teaching faculty members towards using smartphone as a teaching tool

No.	Item	Mean	Standard Deviation
1	Smartphones are useful as a supplementary to teaching.	2.08	0.81
2	Smartphones improve access to my courses and learning material.	1.81	0.72
3	Smartphones help me organize my work better.	2.00	0.85
4	Smartphones enhance easier access to information anywhere and anytime.	1.46	0.658
5	Text messaging via smartphones is useful as an instructional tool in class.	2.31	1.02
6	Shooting videos of lectures allows students who miss class or may not have caught something the first time.	2.42	1.11
7	Smartphones can increase in class participation and elsewhere collaboration between students.	2.43	1.01
8	Smartphones increase communication between the lecturer and the student.	2.12	0.85
9	Smartphones can help students be more prepared for class by easily accessing information before class.	1.97	0.82
10	Smartphones provide students with the opportunity to work at their own pace.	2.19	0.91
11	Smartphones allow students to get access to up-to-date information through the Web and social media.	1.75	0.67
12	Smartphones can green up the classroom by converting as many class materials to digital as possible.	2.18	0.87
13	Smartphones can encourage students to store everything on their smartphones, Tablets, computers, or other device.	1.84	0.67
14	Smartphone features allow users to learn grammar, spelling, pronunciation, and other essential literacy skills.	1.96	0.90
total	General Question: The Perception of teaching faculty members towards using smartphone as a teaching tool	2.08	0.73

**Fig. 7:**
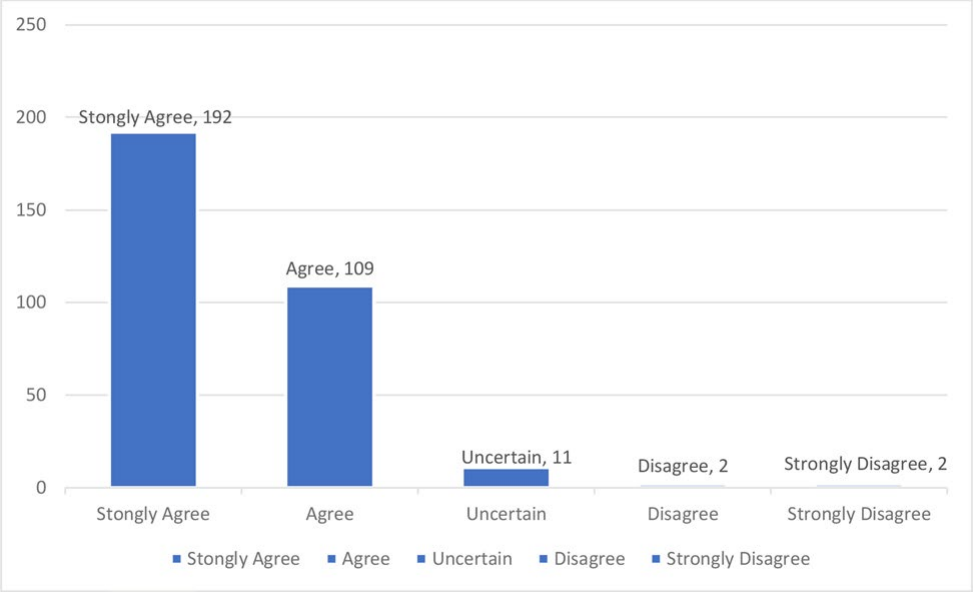
shows faculty results of the question that smartphones enhance easier access to information anywhere and anytime

Table 1 shows us the perception of teaching faculty members towards using the smartphone as a teaching tool. As we can see that most of the faculty members either agreed or strongly agreed to the question that smartphones make it easier to access information anywhere and anytime (Mean 1.46 SD 0.65) this mean interprets that most of the members chose the option strongly agree or agree. This is shown in the Fig. 7 pie chart as well.

**Fig. 8:**
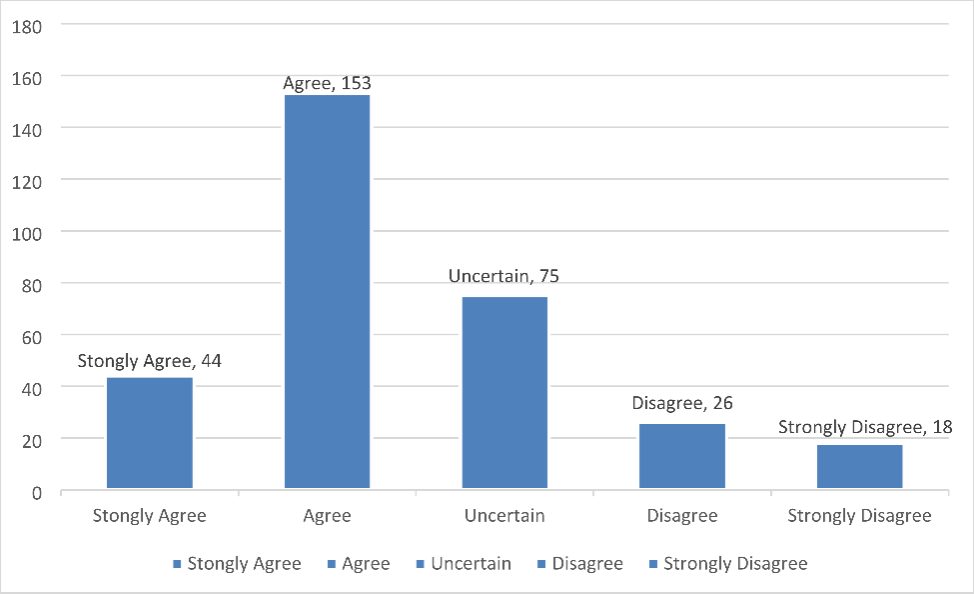
Faculty response to smartphones can increase in class participation and elsewhere collaboration between students

Similarly, the faculty response to the question that Smartphones can increase in class participation and elsewhere collaboration between students was positive with Mean 2.43 SD 1.01. This means that most of the faculty chose agree or remained uncertain about this. This is shown in Fig. 8.

**Table 2:** Statistical results related to faculty members perceptions towards smartphones based demographic variables

Demographic Features		Mean	Standard Deviation	Significance *P* value
1. Title	Professor	2.29	0.97	0.14
	Associate Professor	2.05	0.74	
	Assistant Professor	1.97	0.81	
	Lecturer	2.09	0.83	
	Demonstrator	1.95	0.82	
2. College	College 1	2.03	0.90	0.04
	College 2	2.27	0.70	
	College 3	1.98	0.88	
	College 4	1.81	0.64	
	College 5	2.13	0.89	
	College 6	1.80	0.51	
	College 7	2.11	0.72	
	College 8	2.18	1.00	
	College 9	2.08	0.77	
	College 10	1.47	0.47	
	College 11	2.20	0.76	
3. Departments	Anatomy	1.42	0.00	0.05
	Biochemistry	1.98	0.85	
	Community Dentistry	2.20	0.54	
	Dental Materials	2.46	1.17	
	Medical Education	1.97	0.73	
	Operative Dentistry	1.90	0.72	
	Oral Biology	2.03	0.65	
	Oral Medicine	1.82	0.61	
	Oral Pathology	2.03	0.93	
	Oral Surgery	2.14	0.74	
	Orthodontics	2.02	0.85	
	Pediatric Dentistry	1.88	0.56	
	Pathology	2.39	0.45	
	Periodontology	1.91	0.84	
	Pharmacology	2.96	0.82	
	Physiology	1.64	0.57	
	Prosthodontics	1.81	0.81	
4. Gender	Male	2.08	0.86	0.28
	Female	1.97	0.83	
5. Experience	Less than 5 Years	1.95	0.82	0.27
	5 to 10 Years	2.09	0.83	
	More than 10 Years	2.09	0.88	

Table 2 shows us the perceptions of faculty members perceptions towards smartphones against the demographics. We can see that according to title the results were not significant (p-value 0.14). Similarly, according to the departments it was not significant (p value 0.05). Based on gender, the result was statistically insignificant (p-value 0.28) and similarly in experience as well the results were statistically insignificant (p-value 0.27), However, according to colleges the results were statistically significant (p-value 0.04) which shows that not all colleges are equally utilizing the potential of smartphones.

## Discussion

The Dental faculty members of Khyber Pakhtunkhwa generally had a positive perception of using a smartphone as an educational tool. With the majority of the teachers opting for strongly agree and agree options. There was also consensus regarding easier access to information including courses and learning material, and improved communication between the teachers and students. Furthermore, the results showed us that social media has a very good impact on students regarding keeping them updated about courses and information. This is also cemented by the fact that different brochures are shared online via social media platforms these days and usually students get updates from there. Another growing advantage is of smartphone is the memory. The manufacturers have achieved to put really large memories in smartphones which help the students to save all the information on their smartphones. So the information is readily avaible on their fingerprints. Besides all these advantages that the faculty agreed upon there is another one in the form of language and grammar. There are inbuilt dictionaries and assistive writing softwares in the smartphones that help the students in increasing their vocabulary and enchance their literacy skills.

On the contrary, faculty members were not agreeable regarding the shooting of videos during the lectures for the students who missed the lectures for one reason or another.

There was a significant difference among the different departments of dental program. Some basic sciences departments are not very fond of students utilizing their smartphones during their lectures. They were of the view that conventional lecture was the only way to teach their subject.

Our results are similar to the results of the study conducted by Jabali et al., they investigated the use of smartphones by faculty and using smartphone as a teaching tool in two universities of Palestine [4]. We had eleven dental colleges in our study whereas they only did the study only in two universities. Their sample size was also less as compared to ours, but they used the same questionnaire.

Four of the faculty members reported that they didn’t use a smartphone. This is a very small number, but we need to make sure that both our faculty and students have adequate computer literacy. This was shown in a study conducted in Sweden in 2005 which assessed computer literacy among dental educators and students. They concluded that there was a significant difference found between the competence measured and the year of graduation of the dental educator’s group. However, regardless of the competence the attitudes of the educators regarding the use of information and computer technology were very positive, regardless of their competence, year of graduation or academic position [15]. This is also in line with our study. Most of the attitude of our dental faculty regarding using smartphone and internet for accessing information is positive. Similarly, computer literacy courses should be introduced for both faculty and students in the colleges for them to learn and have a basic idea about using technology to enhance their teaching and learning skills.

In another study, Maureen McAndrew explored the use of social media in Dental education. He concluded that most of the students are millennials and are very fond and avid users of social media, they often reside to these websites for their knowledge and collaboration tools. Dental educators should look into a proper way of integrating these technologies into the dental education, which will bear many fruits [16]. The dental faculty of Khyber Pakhtunkwa also agreed that smartphones enable students to get up-to-date knowledge from social media apps. In another study in Australia, it was concluded that students use a smartphone and social media for their educational activities even though it was not formally introduced as teaching tool in the curriculum.^(1)^ This gives us an idea that the dental faculty of KPK should start working on introducing the smartphone as a teaching tool and prepare some groundwork so that dental education in KPK doesn’t lag behind the rest of the world.

In a study conducted in Saudi Arabia in 2016, Researches concluded smartphones is being used by students and doctors for communication and sharing of knowledge regarding patients but many of them are using without any proper security feature and this can lead to dreadful consequences it the patient confidentiality is breached. In this regard there must be policies and security measures introduced in order to utilize this growing technology safely into the health care sector [17]. This is inline in our studies that teachers are of the view that smartphone is a useful tool but its use should be monitered carefully while implementing it into our healthcare and educational sector.

There are obviously too many barriers to implementing such technology in our society. Not all students can afford a high-end smartphone. And not all colleges are equipped with a high-speed internet facility. So, we need to make some changes in our curriculum to implement these new tools and inclusivity. Given that our faculty members have positive attitudes and they themselves use the smartphones quite frequency for study purpose. We don’t have the attitude problem at least in the dental faculty of Khyber Pakhtunkhwa.

A study conducted by Balan Rathakrishnan in 2021 highlighted the importace of smartphone addiction and quality sleep in university students and its effects on the academic performance. He concluded that poor performance in academics was related to smartphone addiction and poor quality of sleep and such issues must be addressed as we begin to integrate smartphones into our educational setups [18]. Beside these there are many disadvantages highlighted by the literature which include but not limited to addiction to smartphones, distractions they can cause while using them in the class, the constant and up to date notifications can split a student’s attention quite quickly and distract from what they were aiming to learn. Even while using the smartphone notifications from friends can distract the student to avoid or ignore the work at hand and go on to chat with their friends [19].

### Strengths and weaknesses of the study

- The sample size of the study was large enough to represent the whole dental faculty of Khyber Pakhtunkhwa.
- This study presents an opportunity for all the dental colleges to introduce courses on implementing newer technologies into their teaching and learning strategies.
- This study can help push the college administrations to upgrade their facility with the latest hardware to support using smartphones as a teaching tool mainly providing high-speed internet access on the campus. Which is also now a mandatory option by the Pakistan Medical Commission.
- The limitations include that this study was conducted on the faculty of dental colleges. Whereas major decisions about policymaking takes place by the administrative staff of the colleges. The faculty only gives their input, but the main decision is of the administration. Maybe research into the administrations of dental colleges will yield the answers of why we cannot formally introduce courses in which we rely on technology.

## Conclusion

The results of our study concluded that most of the dental faculty members who answered the questionnaire perceived their smartphone as an effective teaching tool to some extent despite that there has been no formal application by the college or university. According to the results of this study, work on the development of such teaching and learning activities must be started that involved using smartphones. This will not only make the student more interested in learning it will greatly benefit them of remote learning and having the access to information at any given time.

## Electronic supplementary material

Below is the link to the electronic supplementary material.

Supplementary Material 1

## Data Availability

Data is not shared publicly because the permission about open public access has not been granted. it is safely stored on a server. But it is available from the corresponding author on reasonable request.

## List of Abbreviations

ASRB: Advanced Studies Research Board.
ERIC: Educational Resources Information Center.
IHPER: Institute of Health Profession Education and Research.
KPK: Khyber Pakhtunkhwa.
BDS: Bachelor of Dental Surgery.
MeSH: Medical Subject Headings.
KMU: Khyber Medical University.
AIMC: Abbottabad International Medical and Dental College.
AMC: Ayub Medical College.
BKCD: Bacha Khan College of Dentistry.
FMDC: Frontier Medical and Dental College.
KCD: Khyber College of Dentistry.
KIDS: KMU Institute of Dental Sciences.
PDC: Peshawar Dental College.
RCD: Rehman College of Dentistry.
SCD: Saidu College of Dentistry.
SBDC: Sardar Begum Dental College.
WDC: Women Dental College.
