# Supplementary material for: “Smartphone as an educational tool” the perception of dental faculty members of all the dental colleges of Khyber Pakhtunkhwa - Pakistan
Source: BMC Med Educ. 2023 Feb 20;23:122. doi: 10.1186/s12909-023-04093-8 (PMC9942358; doi:10.1186/s12909-023-04093-8)
Supplement: Supplementary file 1 — Supplementary Material 1 [file 12909_2023_4093_MOESM1_ESM.docx]

## (Questionnaire/Consent Form)

Please fill this questionnaire to help us to identify instructors’ uses and attitude towards smartphones in the teaching process. The aim of this questionnaire is to obtain information to incorporate new trends of teaching and instruction at undergraduate level. All data provided will remain confidential. By completing this questionnaire, you are indicating your willingness to participate. Your participation is greatly appreciated.

**Section A**

**Demographics and Social Characteristics**

**1- Title**: Demonstrator□ Lecturer □ Assistant Professor □

Associate Professor □ Professor □

**2.College:**

**____________________________________________________________**

**3.Department: ____________________________________________________________**

**4. Gender**: Male □ Female □

**5. Years of experience**: Less than 5 years □ Five to 10 years □

More than 10 years□

**6. Do you use a smartphone? YES NO**

**Section B**

**Lecturers’ attitudes towards using smartphones as a teaching tool**

| **No.** | **Item** | **Strongly**  **Agree** | **Agree** | **Uncertain** | **Disagree** | **Strongly**  **Disagree** |
| --- | --- | --- | --- | --- | --- | --- |
| 1 | Smartphones are useful as a supplementary to teaching. |  |  |  |  |  |
| 2 | Smartphones improve access to my courses and learning material. |  |  |  |  |  |
| 3 | Smartphones help me organize my work better. |  |  |  |  |  |
| 4 | Smartphones enhance easier access to information anywhere and anytime. |  |  |  |  |  |
| 5 | Text messaging via smartphones is useful as an instructional tool in class. |  |  |  |  |  |
| 6 | Shooting videos of lectures allows students who miss class or may not have caught something the first time. |  |  |  |  |  |
| 7 | Smartphones can increase in class participation and elsewhere collaboration between students. |  |  |  |  |  |
| 8 | Smartphones increase communication between the lecturer and the student. |  |  |  |  |  |
| 9 | Smartphones can help students be more prepared for class by easily accessing information before class. |  |  |  |  |  |
| 10 | Smartphones provide students with the opportunity to work at their own pace. |  |  |  |  |  |
| 11 | Smartphones allow students to get access to up-to-date information through the Web and social media. |  |  |  |  |  |
| 12 | Smartphones can green up the classroom by converting as many class materials to digital as possible. |  |  |  |  |  |
| 13 | Smartphones can encourage students to store everything on their smartphones, Tablets, computers, or other device. |  |  |  |  |  |
| 14 | Smartphone features allow users to learn grammar, spelling, pronunciation, and other essential literacy skills. |  |  |  |  |  |
